# The experience of caregivers of people living with serious mental disorders: a study from rural Ghana

**DOI:** 10.3402/gha.v8.26957

**Published:** 2015-05-11

**Authors:** Kenneth Ayuurebobi Ae-Ngibise, Victor Christian Korley Doku, Kwaku Poku Asante, Seth Owusu-Agyei

**Affiliations:** 1 Kintampo Health Research Centre, Ghana Health Service, Kintampo, Ghana; 2 Centre for Global Mental Health, Institute of Psychiatry, Psychology and Neuroscience, King's College London, United Kingdom

**Keywords:** burden, primary caregiver, mental disorders, stigma, Kintampo

## Abstract

**Background:**

Families and friends who give care to people with mental disorders (MDs) are affected in a variety of ways and degrees. The interplay of caregiving consequences: poverty, discrimination and stigma, lack of support from others, diminished social relationships, depression, emotional trauma, and poor or interrupted sleep are associated caregiver burden.

**Objective:**

The burden of care on caregivers of people living with MDs was assessed in two districts located in the middle part of Ghana. Coping strategies and available support for caregivers of MDs were also assessed.

**Design:**

A qualitative study was carried out involving 75 caregivers of participants with MDs registered within the Kintampo Health and Demographic Surveillance Systems. Data were gathered from caregivers about their experiences in providing care for their relations with MDs.

**Results:**

Caregivers reported various degrees of burden, which included financial, social exclusion, emotional, depression, and inadequate time for other social responsibilities. Responsibilities around caregiving were mostly shared among close relatives but to a varying and limited extent. Religious prayers and the anticipation of cure were the main coping strategies adopted by caregivers, with expectation of new treatments being discovered.

**Conclusions:**

Emotional distress, stigma, financial burden, lack of support networks, social exclusion, health impact, and absence of decentralised mental health services were experienced by family caregivers. These findings highlight the need for interventions to support people with MDs and their caregivers. This might include policy development and implementation that will decentralise mental health care provision including psychosocial support for caregivers. This will ameliorate families’ financial and emotional burden, facilitate early diagnosis and management, reduce travel time to seek care, and improve the quality of life of family caregivers of persons with MDs.

The substantial and growing public health burden arising from mental disorders (MDs) across the world has been well documented (1–6). The burden of MDs is estimated as 14% globally (7), with the highest burden in developing countries (8, 9) leading to continued economic burden and suboptimal productivity at the individual and national levels (10, 11). MD convtributes significantly to global disability-adjusted life-years (12).

MDs have considerable negative consequences on the quality of life of patients and their caregivers or friends, particularly in low- and middle-income countries (13). Caregiver burden has been described as the overall physical, emotional, and financial costs of caring for a relative suffering from the medical condition. Caregivers’ experiences encompass distress, stigma, worry, shame, and guilt, but with positive aspects such as caregiver reward (14–16).

In the past decades, most researchers and mental health professionals have concentrated much of their time and efforts on people living with MDs with particular interest on causation and symptomatology (17, 18). There is now the need to focus on caregivers of patients with MDs as well, especially in developing countries where health systems for managing mental health patients and their caregivers are lacking (19, 20). Unavailability of proper health systems in developing countries affects quality of life, thus worsening mental health diagnosis and treatment, as well as documenting disabilities among community members with MDs and worsening caregiver burdens. This study involved caregivers of patients diagnosed with serious MDs such as persons diagnosed with schizophrenia.

The role of caregiver in patient management is well documented for chronic ill health such as cancers and physical disability in developed countries (18, 21–23) as well as HIV in developing countries (24, 25). In Ghana and other African countries, wide family networks including church societies provide support to individuals or groups of people when social problems such as death (26, 27) or disasters (28) occur. Such social support systems do not exist, however, for illnesses such as mental health, which is widely stigmatised. Therefore caregivers of mentally ill health patients have no choices but to carry their physical, emotional, spiritual, and financial needs solitarily (29–31). There is therefore the need for better understanding of caregiver experiences in providing care for their relatives affected by MDs as this will help health service providers and social service networks to better understand the needs of caregivers when targeted caregiver interventions are planned.

This study assessed the burden of caregiving for relative(s) living with MDs in two districts located in the middle part of Ghana that are predominantly rural. The characteristics of caregivers of people living with mental illness and their coping strategies and available support including community support towards the care of people living with MDs are described.

## Methods

### Study design

Qualitative interviews (in-depth interviews [IDIs] and focus group discussions [FGDs]) were undertaken between January and June 2010 using purposively sampled caregivers of community members living in two adjoining districts of Ghana: Kintampo North and South Districts.

### Study area

The studied districts, with a resident population of about 140,000, are located in the Brong Ahafo Region of Ghana; they are predominantly rural. Two government hospitals, two private hospitals, four health centres, one private clinic, 25 community health planning services zones, and two private maternity homes form the health facilities in the studied area. Only one of the hospitals provides mental health services in the study area. These services are provided by two community psychiatric nurses, who are supplied with psychotropic medications for routine care of mental health services. Only patients with valid Ghana National Health Insurance Scheme are able to access the services. The nearest access to a psychiatrist is the Brong Ahafo Regional Hospital, Sunyani, which is located about 136 km away from the study area. There are neither social support programmes nor counselling services for patients with mental health disorders and their caregivers.

### Participant identification and selection

The study involved residents in the two Kintampo Districts who are registered as part of the Kintampo Health and Demographic Surveillance System (KHDSS). The KHDSS provides longitudinal data of residents in the study area (32–34) and maintains a register of residents diagnosed with mental and neurological disorders. This case register is updated every 3 to 6 months and allows for monitoring of demographic changes, for example, mortality, prevalence, and incidence of mental illness over time in the catchments area. It also allows for easy location and follow-up of patients during outreach programmes. New patients who are identified in the update rounds are recorded in the MD cases book. Referrals from ongoing studies in Kintampo Health Research Centre (KHRC) and by community-based key informants are also updated in the MD case register. In total, 700 primary caregivers of the same number of patients with different kinds of mental and neurological disorders were registered in the mental health case register at the time of this study, The diagnoses of MDs were made by the community psychiatric nurses using the *International Classification of Diseases*, 10th edition, primary care version for mental disorders (*ICD-10*, PCV). There were in total 75 primary caregivers of patients with serious MDs (such as schizophrenia) registered on the KHDSS database; they were all included in this study.

Participants were identified in their homes and asked for consent to be part of the study. We did not encounter any refusals from the caregivers to participate in the study.

### Data collection

IDIs and FGDs were conducted among caregivers of persons documented in the case report book, as living with mental health problems. Both IDIs and FGDs were used to compliment as well as validate the generalisability of the responses solicited from the qualitative interviews, a design that allows us to explore individual level as well as the group contextual issues about caregiver burden (35). An interview guide was designed and used to moderate the FGDs, with each FGD group composed of both male and female caregivers, with group membership between 8 and 12. There were three facilitators including one moderator and two note takers. The IDIs were conducted in the participant's homes, whereas the FGDs were conducted in the central locations of the communities which were involved in the study.

Seventy-five interviews and six FGDs were conducted among primary caregivers of persons living with MDs to explore the experiences or contextual issues relating to caregiving for people with mental illness.

The levels of burden associated with caregiving were explored qualitatively for each patient. Themes bordered on caregiving burden including participant's experiences of caregiving inventory and ways of coping as well as the available support for caregivers of people living with MDs. Basic demographic information such as age, sex, marital status, occupation, educational level, and place of residence of the selected caregivers was documented as part of this study. The impact of the caregiving role on caregivers’ finances, emotion, social relations, time, and health were assessed by trained psychology graduates with bachelor's degrees. In other words, we explored how caregiving for persons with MDs affected family finances, and the psychology and health of the caregivers.

### Data analysis

The IDIs and FGDs were recorded digitally and transcribed verbatim. *A priori* and emergent themes were coded and analysed with MAXQDA version 10 (VERBI Software, Berlin, Germany). Interviews were conducted in the widely spoken local language (Twi) and were transcribed verbatim, translated into English, and back translated into Twi to ensure that the statements used during the discussions were maintained.

The framework analysis approach was used (36). This is a case- as well as a theme-based approach of qualitative data analysis which displays matrix, and reduces data through summarisation and synthesis for easy comprehension. The original data link is also retained with this approach. A coding frame based on the objectives of the study was developed. The overall analysis was done paying attention to concordant and contrasting views. As part of this approach, an a *priori* analytical framework was developed, using themes based on the aims of the study, and emergent themes were added to this framework as analysis proceeded. New themes, which were not part of the predetermined objectives of the study, but came out from the interaction with the study participants, were included in the final analysis.

### Ethical approval

Approvals were provided by the KHRC Scientific Review Committee and the KHRC Institutional Ethics Committee (FWA: 00011103). Anonymity and confidentiality was ensured throughout the conduct of the study. Personal identifiers were replaced by reference values during data analysis. Completed questionnaires were properly kept under locks. Each participant in the study provided a signed/thumb-printed informed consent. These consent forms were countersigned by witnesses of study participants and a research team member.

## Findings

We present here findings of the study which included a description of the participants and the experiences of caregivers of people with MDs. There were five themes that came out from the final analysis including, stigma and emotional burden, burden on time, economic burden, available support for caregivers, and the coping strategies adopted by caregivers.

### Demographic characteristics of the study participants

A total of 75 primary caregivers who were selected, consented and were interviewed. Most of the caregivers were females, 56% (42/75), and 60% were aged between 35 and 64 years. More than 65% (49/75) of the caregivers had no formal education and the majority had no gainful occupation (Table 1).

**Table 1:** Demographic characteristics of the primary caregivers of patients with MDs in the study area of the Kintampo Health and Demographic Surveillance System, 2010

Exposure	Frequency (*N=*75)	%
Sex		
Female	42	56.0
Male	33	44.0
Age group		
16–34	15	20.0
35–64	45	60.0
65 +	15	20.0
Education		
No formal	49	65.3
O/A level	23	30.7
Tertiary	3	4.0
Occupation		
None	24	32.0
Farming	21	28.0
Business	12	16.0
Casual	18	24.0
Marital status		
Single	15	20.0
Married	45	60.0
Widowed/divorced	15	20.0
Relationship to patient		
Parent of patient	32	42.7
Sibling	24	32.0
Daughter/son	13	17.3
Other relatives	4	5.3
Partner	2	2.7

### Demographic characteristics of the patients with MDs

More than 53% of cases were females and over 60% were aged between 21 and 45 years (Table 2).

**Table 2:** Demographic characteristics of patients with MDs in the study area of Kintampo Health and Demographic Surveillance System, 2010

Exposure	Frequency (*N=*75)	%
Sex		
Female	40	53.3
Male	35	46.7
Age group		
1–10	3	4.0
11–20	9	12.0
21–45	46	61.3
46–64	15	20.0
65 +	2	2.7
Occupation		
None	59	78.7
Farming	6	8.0
Teaching	1	1.3
Casual	7	9.3
Marital status		
Single	57	76.0
Married	7	9.3
Long-term partner	1	1.3
Widowed/divorced	10	13.3

## Experiences of caregiving of people with MDs

### Stigma and emotional burden

An emotional distress in taking care of the mentally ill was frequently reported. Most caregivers reported that almost all the time they found themselves thinking about their relatives who are sick and the embarrassment that sometimes accompanied. They are made to perform rituals that are a source of worry and stress to them. Some caregivers described these experiences in the following way during the FGDs and the IDIs:

It hurts me seeing how he is and being like that among us. It hurts me a lot. I am actually stressed about that. (A female respondent at Community AS FGD)

When I sleep, I begin to think about my mother's condition, it may happen that as we sleep with her, she may just die. I sometimes think about that. (A female respondent at Community KP FGD)

Some respondents indicated how they are unable to sleep because of the behaviour of their mentally disturbed relatives:

He was very sick and we can't afford to sleep, he will cry till day break. I am always afraid of how he behaves because I don't know the kind of sickness that he is suffering from. (A male respondent at Community KP IDI)

For our health, we will take it like that, I don't sleep. He does not allow me to sleep. He will talk till day break. (A male respondent at Community XA FGD)

Some of the care givers indicated how their daily duties are affected because of the actions of their mental health patient that they are taking primary responsibility for.

It affects me anyway. I will be crying anywhere I go whether to the farm or anywhere. When I am working I will be crying. (A female respondent at Community XA FGD)

I am depressed anyway and sometimes afraid. (A male respondent at Community XA IDI)

Some caregivers reported that their social relationships are negatively affected because they are not in the position to move and interact freely with other colleagues. This connotes perceived societal stigma against people with mental problems. One respondent said:

Because of this problem, instead of you to move on with your colleagues, you can't and it becomes a problem for you since you cannot go near them. (A male respondent at Community DD IDI)

Caregivers undergo emotional disturbance when their family members become aggressive or abusive to them or their friends. This is particularly worrying to caregivers when the aggression is targeted at new friends who visit their homes for the first time. This is demonstrated as below:

How my mother is now, if anybody that you respect is coming to the house, that is the time she will be insulting you. Anybody who speaks to her, she starts to fight them.

Day in day out, she will pick people's items and they will report to me and I will be pleading. (A female respondent at Community DD FGD)

It is difficult because she disturbs everybody. We are staying with our father and she could disturb our father till we all become sad because she could sometime try to raise the hand on our father. There is a problem. (A female respondent at Community MK FGD)

### Burden on time

Most caregivers spend most of their time taking care of their relatives who are suffering from MDs. Some of the responsibilities of the caregivers include bathing, seeking medical attention including visits to the traditional herbalist, security, preparing meals and feeding them when necessary, and assisting them during nature's call, among others. Some caregivers even claimed that they suffer more than those who are ill.

All the time. That is why I said that the sick person does not suffer but you the caretaker have to get time, all the time for him/her. (A female respondent at Community AA FGD)

The duration spent in caring for the patients varied but some caregivers explained that as long as the patient's sickness is not cured, they had no choice but to continue caring for him/her. There was a sense of frustration in the amount of time devoted to caring for the patients. One respondent sees death of the patient as an end to the time spent in caring for the patient.

All the time because if the sickness is not gone, you are also not relieved. Always, you are burdened with sufferings. If the person is not healthy, you do not also rest. The person is not dead too, can you say you want to rest? (A female respondent at Community DD FGD)

I spend a lot of time. How she is behaving, if you are there, you start thinking and become worried sometimes you wake up and look disturbed because she is in the room and will not get out. (A male respondent at Community KP IDI)

### Economic burden

A majority of the caregivers were not employed, or employed as peasant farmers or casuals (Table 1). In taking care of their relatives, some caregivers would work and/or sell their belongings as well as other resources such as personal clothing or family livestock until their belongings were exhausted. Caregivers are also involved in menial jobs to take care of themselves and their relatives. Some of the caregivers are not able to work because they have to stay at home and take care of the sick person. Caregivers who are unable to work depend mostly on other distant relatives for financial support. Some caregivers expressed hopelessness in making economic gains while caring for their ill relatives. One of them indicated:

The problem is if I grow crops like rice, I harvest and process it. Looking at the condition of my child, I sell all and if I hear somebody saying he/she can take care of my child …. I will do that (pay for the cost of illness) until all my money is exhausted. (A male respondent at Community DD FGD)

Other caregivers described the lack of financial support and that there is no external support even if they wanted to talk about it.

Eh! (Expression of extreme shock) For the financial aspect, I can't say much. If I even say it, there is no helper from anywhere. (A female respondent at Community XA IDI)

One caregiver indicated that caring for someone living with a mental disorder has a negative impact on her finances and emotional health.

Because of the condition, my work is not moving on well. I had to stop and go and look for some money elsewhere so that my siblings will continue their education … It also becomes a problem, you pull and nothing comes. In all, there is a problem, because what you need to bring to the house to improve the home is hindered. Everything is scattered. (A female respondent at Community DD FGD)

### Available support for caregivers

There was no reported support for caregivers of people living with MDs. The only mentioned support was for the patients with MDs but not their caregivers.

Community support in caring for patients with MDs was lacking. Almost all the study participants were unanimous on the fact that there is no external or community involvement in taking care of the mentally ill. Immediate family members such as father/mother/siblings are the ones that sometimes extend support for the ill person. Some of the respondents indicated the following:

I don't get any help from anywhere. If I tell you that I get something from my family, it is a lie. (A female respondent at Community AA IDI)

Eh! I unless you go to a known person to help. (A female respondent at Community KP IDI)

One caregiver was, however, of the opinion they mostly need support for their relations with MDs and not directly for themselves. She said:

What we need is assistance for those we are taking care of to reduce the burden on us. (A male respondent at Community DD IDI)

A few caregivers who believe that the cause of their relatives’ MDs was not spiritual had visited health facilities and said they have received support in the form of a supply of drugs from health workers. Apart from that there is no other support received. There was no evidence that caregiver burden was different from relatives who access care regularly. Caregivers who sought health care for their relatives sometimes travelled about 70 km to access psychiatric care where supply of medication was also erratic.

The reasons for lack of support for caregivers were explored further. The caregivers indicated that there was general poverty in the community; nobody therefore had surplus money or resources to offer. Other factors responsible for the lack of support for caregivers of MDs are stigma and lack of empathy. Some of the respondents demonstrated these in the following conversation when asked about the support for caregivers of people living with MDs:

I said initially that, you may think your brother has and you are crying that he did not give but he does not have. So I will not depend on him for help. They do not have. (A female respondent at Community AA IDI)

I know my siblings do not have anything to help me. Since the person does not have, I cannot rely on him that he/she did not help me. If the person has, you can rely on but he/she does not have. (A male respondent at Community AA IDI)

The caregivers also indicated that they will need support in the form of education so that mental illness is not misconstrued and not stigmatised. One caregiver said:

We need support from the health workers, they should expand it and the education should continue. It will help others. For us here, we have some of the sick people in our houses so if I go and see that of my brother, I can even lift him but somebody who does not have such people and they do not know about it, will not even touch. Even if the person gets up, such a person will have to make a sacrifice before he/she will pass there. (A female respondent at Community AS FGD)

### Coping strategies adopted by caregivers

Caregivers adopted various coping strategies to deal with the burden of taking care of their mentally ill relatives. Some of the coping strategies include use of prayers, hope for a miracle, and anticipation of a new treatment regime.

Most of the time caregivers relied on prayers offered by pastors and other divine instructors, hoping for miracles as well as new treatment regimens as a coping mechanism in dealing with the stress of caring for their sick relatives. Some of the prayers are usually conducted at prayer camps (centres organised by Christians that serve as places where many people seek healing and deliverance. Prayers and fasting are observed by patients and their caregivers for the divine healing of patients). Some caregivers resort to prayers after a long search for appropriate medications.

That is the movement [referring to movement in search for medication], which I am tired of roaming. Now God has shown us the way that I should send her to the prayer camp. Now she is being taken care of by the priest and by God's grace she is now better. (A female respondent at Community XA IDI)

## Discussion

The unmet need of people living with MDs all over the world has a spillover effect on those caring for them (37, 38). Not much work assessing the experience of caregivers of people living with MDs has been reported even though this is important for service planning and delivery. However, the need for attention for those who really care for people with MDs is as important as addressing the problem holistically (39).

This study revealed psychosocial, emotional, economic, and physical challenges caregivers undergo in caring for their relatives saddled with mental illness. The findings point to problems such as: stress, lack of support or social service provision, poverty due to their inability to work full time resulting in financial difficulties, and general societal stigma of living with someone with mental illness. The findings from this study are similar to several other studies conducted in the past in relation to the challenges caregivers go through when caring for persons with MDs (40–42).

The majority of caregivers were females who were psychologically distressed as a result of the enormous responsibilities they shoulder on MDs. There was a strong sense of distress and hopelessness among the caregivers. The finding in this study is consistent with a previous study in Malaysia among family caregivers of mental patients that documented half of the caregivers were psychologically distressed (39) and that a relative with psychological problems invariably affects the caregiver (43). Our study also found that some of the caregivers reported negative effects on their emotional health, social activities, and leisure time, and more than half reported adverse effects on family relationships. This finding is also comparable to previous studies, which reported that caregivers of mental patients are emotionally stressed and their social activities diminish (44, 45) due to their caregiving commitments.

In this study, financial burden was one of the major challenges faced by caregivers. This finding is similar to that found in United States of America (46), Ghana (47), and in other rural African settings such as in Ethiopia (48) and Nigeria (49). In Nigeria, caregivers rated financial matters as posing greater burden than other areas such as burden of disruption of family routine and interaction, social stigma, and subjective distress (49). A similar study conducted in the United Kingdom reported that caregivers have higher levels of unmet needs when they have high levels of financial burden (50). In our study, a majority of the caregivers lived in rural areas and were either unemployed, peasant farmers, or are involved in petty. It is likely that in rural areas where household income is low, caregivers undergo an extra financial burden due to the competing needs of their MD relative, and their inability to be gainfully employed. We recommend that caregivers be provided with financial support in the form of soft loans to set up businesses or be provided with living allowances as part of government social interventions.

Our study results demonstrated perceived impact of caring for patients with mental health on caregivers’ physical and emotional health. This observation is similar to those reported in other studies in Nigeria (51), Taiwan (52), and Norway (53). Their poor physical and emotional health may result from lack of time for other supportive activities such as recreational activities including games. The poor emotional drive may lead them to depression that will have adverse impact on their relatives with MDs. In this study, caregivers reported spending so much time in caring for their relations with MDs that, they hardly find time to attend to any other social needs commitments.

Apart from minimal family support, there was no other support for caregivers of people living with MDs, even though caregivers accepted their responsibility of caring (54). This is similar to most developing countries were support for caregivers and their patients are inadequate (40). There is the need for government to extend the social support to caregivers of people living with MDs, just as is being done to people who are physically or mentally disadvantaged in society. Currently, there is no such support for caregivers of people living with MDs in the study area. There is the need for strong institutional professional support for caregivers of patients with MDs to reduce their burden as demonstrated by Reinhard et al. (55).

In our study area where psychiatric service is lacking, caregivers rely on prayers for divine intervention in the form of miracles to cope with the burden of taking care of their sick relatives. This coping mechanism of prayer is similar to that found in a study in the United Kingdom where there are better psychiatric services (56). Irrespective of availability of psychiatric services, prayers seem to provide a sense of hope for caregivers that may help them to cope with caregiver burden.

Our study included caregivers of patients with severe MDs who are likely to have negative symptom behaviours. In previous studies conducted in 19 countries of the WHO World Mental Health surveys, the burden of caregivers has been found to be worse if their relatives with MDs had depressive behaviours (55), negative symptom behaviours (57), or embarrassing behaviour (58, 59). This suggests that the burden of caregivers found in this study may be as a result of the severity of their relatives’ MDs. It is likely that their burden may be less if their relatives are treated and have fewer negative symptoms.

## Conclusion

Caregivers of people with MDs experience various psychological and emotional, social, physical, and economic challenges. There was no reported external support for these caregivers who were equally suffering from mental health problems. Professional assistance in the form of counselling; public awareness of mental illnesses in general; and economic support by all stakeholders including the government, private sector, and non-governmental organisations are vital in addressing these challenges faces by caregivers of people living with MDs.
